# Combination of nanoparticle green tea extract in tris-egg yolk extender and 39 °c thawing temperatures improve the sperm quality of post-thawed Kacang goat semen

**DOI:** 10.1590/1984-3143-AR2022-0025

**Published:** 2023-01-16

**Authors:** Imam Mustofa, Suherni Susilowati, Tri Wahyu Suprayogi, Yudit Oktanella, Djoko Agus Purwanto, Adeyinka Oye Akintunde

**Affiliations:** 1 Division of Veterinary Reproduction, Faculty of Veterinary Medicine, Universitas Airlangga, Surabaya, East Java, Indonesia; 2 Department of Veterinary Reproduction, Faculty of Veterinary Medicine, Brawijaya University, Malang, East Java, Indonesia; 3 Department of Pharmaceutical Chemistry, Faculty of Pharmacy, Universitas Airlangga, Surabaya, East Java, Indonesia; 4 Department of Agriculture and Industrial Technology, Babcock University, Ilishan-Remo, Ogun, Nigeria

**Keywords:** catalase, frozen-thawed sperm, small holder and farm, small ruminant, sperm function

## Abstract

Kacang goats are small ruminants produced by low-income households in smallholder and farm to reduce poverty and prevent undernutrition. Studies to find a cryopreservation protocol for Kacang goat semen are expected to multiplication of genetically superior animals selected by the paternal lineage. This study evaluated the effect of thawing temperature and supplementation of the green tea extract nanoparticle in skim milk-egg yolk (SM-EY) extender on post-thaw sperm quality of Kacang goat semen. Six ejaculates of Kacang goat were diluted in SM-EY supplemented or not (control group) with 0.001 mg/mL NPs GTE. The diluted semen was packaged with 0.25 mL straws (insemination dose: 60x10^6^ sptz/mL) and cryopreserved. Then, six samples of the control group and NPs GTE groups were thawed at 37°C or 39°C sterile water for 30 s and submitted to sperm quality evaluations. The sperm viability, motility, and intact of the plasma membrane (IPM) were higher (p<0.05) in NPs GTE group than control group. In contrast, the NPs GTE group presented lower (p<0.05) malondialdehyde levels and sperm DNA fragmentation (SDF) compared with the control group. The catalase levels were not significantly different (*p* > 0.05) between the control and NPs GTE groups. Thawing at 39°C resulted in higher (p<0.05) sperm viability, motility, and IPM than thawing at 37°C. However, thawing at 39°C group presented lower (p<0.05) malondialdehyde levels compared with thawing at 37°C. SDF and catalase levels were similar (p>0.05) between thawing at 37°C and thawing at 37°C. In conclusion, supplementation of 0.001 mg/mL of NPs GTE in SM-EY extender and thawing temperature of 39°C resulted in a better quality of frozen-thawed Kacang goat semen.

## Introduction

Kacang goats are small ruminants usually reared by low-income households in smallholder and farm. Artificial insemination (AI) techniques can increase ^goats' population^ and genetic quality. Unfortunately, frozen semen of Kacang goat is not commercially available. Several studies conducted to date have not found appropriate protocol for freezing goat semen and the right temperature for thawing. The main problem is the susceptibility of Kacang goat sperm to cryodamage (Susilowati et al., 2021a) than that of the other farm animlas, such as Simmental bull, ram, and Ettawa goat (Mustofa et al., 2021). Based on a lipidomics study, the double bonds in the PUFAs are susceptible to free radical attack and induction of lipid peroxidation (Evans et al., 2021). Highly unsaturated acyl chains are related to spermatozoa's susceptibility to reactive oxygen species and lipid peroxidation. The differences in the sperm plasma membrane lipid composition among species explain different responses during semen cryopreservation (Gautier and Aurich, 2022). Researchers had not yet obtained satisfactory results of post-thawed Kacang buck semen quality. This is presumably due to the high PUFA content in the sperm membrane of Kacang buck sperm, thereby more sensitive to cryodamage. Post-thawing of Kacang goat semen, which froze in SM-EY diluter without any antioxidant, resulted in more than 60% dead sperm (Susilowati et al., 2020), which did not meet the requirement for AI (INSA, 2014). The reduction of live sperm after a freezing protocol is usually caused by an increase of reactive oxygen species (ROS) that can damage the polyunsaturated fatty acids (PUFA) of sperm plasma membranes, increase MDA levels (Fujii and Imai, 2014), reduce sperm viability sperm motility (Peris-Frau et al., 2020), and sperm DNA fragmentation (Mustofa et al., 2021; Ribas-Maynou and Benet, 2019). Our previous study showed that ethanol extract of green tea leaves increases the semen quality and reduces mtDNA mutation of post-thawed Kacang goat sperm (Mustofa et al., 2021). The small size of nanoparticles resulted in a more excellent surface-to-volume ratio (Rakib-Uz-Zaman et al., 2022) makes it easier to reach the outer cell membrane, interact with the extracellular matrix (Behzadi et al., 2017) and penetrate into the sperm cell (Falchi et al., 2018). Other properties of NPs, such as reactivity, surface area, surface charge, and binding properties, contribute to optimizing the freezing protocol (Jeevanandam et al., 2018). These properties allow the chemical and physical reactions of NPs more efficient than microparticles (Joudeh and Linke, 2022). Therefore, NPS GTE antioxidants are expected to work effectively to counteract ROS due to the freeze-thawing process.

Sperm thawing is needed to return the sperm cell to its physiologic temperature at 37°C and reactivate its metabolism (Borah et al., 2015). An usual duration and temperature used for sperm thawing are 30 s at 37ºC (INSA, 2014). The body temperature of a goat is approximately 39°C with small variations (Hereng et al., 2019; Sejian et al., 2021), including the temperature of the vagina when the goat is in heat (Santoso et al., 2014). Several studies reported the temperature and duration of thawing at 35°C for 60 s, 37°C for 30 s, 75°C for 9 s (Borah et al., 2015), 60°C for 7 s, 38°C for 60 s (Penitente-Filho et al., 2014), 39° C for 120 s, and 50°C for 30 s (Nicolae et al., 2014). Our field experience used a fixed thawing duration for 30 s at 37°C. Therefore, based on previous reference studies, this study attempts to increase the thawing temperature to 39°C. However, the appropriate extender for freezing Kacang goat semen has not been developed. Likewise, the optimum temperature for thawing frozen semen as a protocol for implementing AI in the field has not been determined. Therefore, this study determined the influence of nanoparticles of green tea extract (NPs GTE) supplementation in SM-EY extender and different thawing temperatures on post-thawed Kacang goat semen quality (viability, progressive motility, IPM), MDA levels, SDF, and catalase levels.

## Methods

This study was performed in 24 April - 28 December 2021 at the AI Center, Airlangga University, in the Tanjung village of Gresik District, East Java, Indonesia. It is located at coordinates 7° 19′ 25′′ S and 112° 32′ 54′′ E.

## Ethical approval

The procedure of this study has been examined and approved by the Animal Care and Use Committee of Airlangga University with reference number 520/HRECC.FODM/VII/2021.

## Nanoparticles GTE preparation

The green tea leaves were extracted using ethanol solvent as conducted in the previous study (Susilowati et al., 2021b), and the freeze-dried GTE was stored at −20°C until further use. Nanoparticle formulation of green tea was conducted using the chitosan-coated magnetite method. First, 0.5 mL of GTE was added to 0.5 mL chitosan solution at pH 5.0. Next, the mix was homogenized using a vortex for 20 s, after which 0.03% triphenyl phosphate solution was added and homogenized again for 20 s (Sulistyo et al., 2017). The particle size of NPs GTE was measured by a Zetasizer Nano ZS (ZEN 3600, Malvern Instruments Ltd., UK).

## Experimental animals

This study used three Kacang bucks aged 2 to 3 years, weighing 35 to 40 kg. The goats were fed with approximately 4 kg forage crop and 3.5 kg animal feed concentrate (16% to18% crude protein) daily with drinking water *ad libitum*. Semen was collected using an artificial vagina twice a week to obtain six ejaculate samples processed into frozen semen. The ejaculates were pooled to eliminate the individual variation.

## Skim milk-egg yolk extender

Skim milk powder (Merck 115338) 10 g was added in 100 mL distilled water followed by heated to 92°C-95°C for 10 min, then cooled to 37°C. Egg yolk (derived from laboratory chicken eggs) 5 mL was added to 95 ml of skim milk solution. A 1 I.U./mL penicillin (Meiji Seika Pharma, Tokyo, Japan) and 1 mg/mL streptomycin (Thermo Fisher Scientific, Singapore) was then added (Susilowati et al., 2021b). The aliquot was divided equally into two portions: the control group (SM-EY extender without NPs GTE supplementation) and the treatment groups (with 0.001 mg NPs GTE /mL SM-EY extender). The dose NPs GTE in SM-EY diluter was based on a previous study (Mustofa et al., 2021).

## Frozen semen

The first dilution of semen into the extender was conducted at 25°C (laboratory room temperature). Each group of the SM-EY extender was divided equally into two volumes portion. The first volume was added to fresh semen to reach 480x10^6^ spermatozoa/mL. The second volume was added with glycerol to reach 16% (w/v), then added to the first mixture to obtain 240 x10^6^ spermatozoa/mL. The extended semen was cooled from 25° to 5°C for 1 hour, then filled in 0.25 ml French straws (I.M.V., France) at 60 x10^6^ sperm/straw and sealed. The filled straws were placed on steel racks (Cooltop, Minitube) held in liquid nitrogen vapor for 10 min (the temperature decreased from 5°C to − 140°C), and stored in liquid nitrogen (−196°C) for seven days before being evaluated (Susilowati et al., 2020).

## Sperm quality evaluation

Evaluation of semen before freezing was performed at the post-equilibrated step on sperm viability, motility, and IPM. Meanwhile, MDA, SDF, and catalase measurements before feezing were not performed for technical reasons. Semen must be frozen immediately; whereas, the measurements of MDA, SDF, and catalase require varying amounts of time, respectively. Post-thawed sperm quality evaluation was conducted as follows. The straws were thawed at 37°C or 39°C sterile water for 30 s, for semen quality evaluation. Sperm viability, sperm motility, IPM, MDA levels, SDF, and catalase level were evaluated (Susilowati et al., 2020).

## Viability

A drop of sample semen and nigrosine (Sigma-Aldrich) was mixed and smeared on a glass slide, then dried over a bunsen flame. A hundred sperm cells were classified as viable and nonviable sperm under a light microscope (Olympus Bx-53, Shinjuku-ku, Tokyo, Japan) at 400x magnification. The live sperm appeared with transparent heads, whereas the dead sperm were red-colored (Susilowati et al., 2020).

## Motility

Sample semen of 10 µL was mixed homogenously with 1 ml of 0.9% (w/v) NaCl solution, then dropped on a glass slide and covered. The number of progressively motile sperm was counted for 100 sperm in 400× magnification (Susilowati et al., 2020) under a light microscope (Olympus BX-53, Shinjuku-ku, Tokyo, Japan) equipped with Linkam Warming Stages at 37°C-38°C (Meyer Instruments, Houston, Texas, USA**)**.

## Integrity of the plasma membrane

A 0.1 mL of semen sample was added to hypoosmotic solution 1 mL then incubated at 37°C for 30 min. A hypoosmotic solution contained 7.35 g of sodium citrate dihydrate (Sigma-Aldrich) and 13.52 g of fructose (Sigma-Aldrich) dissolved in 1 L of distilled water. The sperm IPM was assessed for 100 sperm under a light microscope (Olympus BX-53, Shinjuku-ku, Tokyo, Japan) at 400× magnification. Sperm with intact plasma membrane showed a curved tail, whereas those with a damaged plasma membrane showed a straight tail (Susilowati et al., 2020).

## Malondialdehyde (MDA) levels

Malondialdehyde levels in the semen were determined using the thiobarbituric acid (Sigma-Aldrich) method. Semen sample100 µL, malondialdehyde kits containing 0, 1, 2, 3, 4, 5, 6, 7, and 8 µg/mL of malondialdehyde were added to 550 µL of distilled water, and 100 µL of 20% trichloroacetic acid respectively. The mixtures were homogenized for 30 s, added with 250 µL of 1 N HCl, homogenized, added with 100 μl of 1% sodium thiobarbiturate, and homogenized again. The final mixture was centrifuged at 28 G for 10 min, and then the supernatant was incubated in the 100°C water bath for 30 min and left at room temperature. Color absorption was determined in a spectrophotometer (Thermo Fisher Scientific) at a wavelength of 533 nm. The malondialdehyde levels (ng/mL) were obtained based on sample’s absorbance values extrapolated against the standard malondialdehyde curve (Susilowati et al., 2020).

## Sperm DNA fragmentation

Acridine orange staining was used for SDF assessment. A drop of semen sample was placed on a glass slide, smeared, air dried, and fixed in 96% ethanol at 4°C for 30 min. Furthermore, the slide was hydrolyzed in 0.1 N HCl at 4°C for 5 min, rinsed with distilled water twice, and then stained with 0.05% toluidine blue (Sigma-Aldrich) for 10 min. The slide was washed in distilled water, dehydrated using t-butanol (Sigma-Aldrich), and cleaned twice with xylol (Sigma-Aldrich). The percentage DNA fragmentation was examined on 100 sperm under a light microscope (Olympus BX-53, Shinjuku-ku, Tokyo, Japan) at 400× magnification. The head of sperm with intact DNA was yellow-colored, whereas the head of sperm with fragmented DNA was green-colored (Susilowati et al., 2020).

## Catalase levels measurement

The H_2_O_2_ phosphate-buffered saline (30 mM) 1 ml was added with 2 mL semen sample (came from 8 straws) at room temperature against a blank containing the enzyme solution. The buffered H2O2 solution (30 mM) made of 0.34 ml 30% H_2_O_2_ was diluted with fresh phosphate buffer (50 mM, pH 7). The UV spectrophotometric absorbance method at 240 nm wavelength measured the catalase activity (Hadwan, 2018).

## Data analysis

This study was conducted with a completely randomized design. The pool of semen was randomly divided into two groups of extenders. Observations were made on samples taken randomly from each group for six replications, respectively. Statistical analysis was performed using Statistic Package and Service Solution (SPSS) software v.23 (IBM Corp., Armonk, NY, USA). Data were subjected to analysis of variance and to Tukey *post-hoc* test at a significance level of 5%.

## Results

The mean ± standard deviation particle size of NPSs GTE was 208.00 ± 26.33 nm. The mean values ± standard deviation of the semen evaluation are: volume 1.48 ± 0.10 mL, pH 6.33 ± 0.52, sperm concentration 1956.00 ± 4.38 x10^6^/mL, viability 86.17% ± 0.98%, and progressive motility 82.50% ± 2.74%.

The supplementation of green tea extract nanoparticles (GTE NPs) in the SM-EY extender increased (*p* < 0.05) sperm viability, motility, and IPM (Table 1) and decreased (*p* < 0.05) MDA levels and DNA fragmentation of post-thawed sperm. However, the catalase levels were not significantly different (*p* > 0.05) between groups (Table 2).

**Table 1:** The effect of nanoparticle green tea extract (NPs GTE) in skim milk-egg yolk (SM-EY) extender and different thawing temperatures on sperm viability, motility, and IPM of Kacang buck sperm.

**Parameters**	**Group**	**Before freezing**	**Thawing temperature**	
			**37°C**	**39°C**
Sperm viability (%)	Control	67.17 ± 1.83^aC^	30.50 ± 1.22^aA^	36.17 ± 1.17^aB^
	NPs GTE	73.17 ± 2.14^bC^	34.83 ± 1.17^bA^	42.67 ± 1.75^bB^
Sperm motility (%)	Control	62.50 ± 2.74aC	25.83 ± 2.04aA	30.83 ± 2.04aB
	NPs GTE	69.67 ± 0.82bC	29.67 ± 0.8 bA	35.17 ± 0.41bB
IPM (%)	Control	65.00 ± 2.83aC	30.67 ± 2.07aA	35.67 ± 1.51aB
	NPs GTE	74.67 ± 1.03bC	35.00 ± 0.89bA	41.67 ± 1.51bB

Control: SM-EY extender, NPs GTE: addition of nanoparticles green tea extract 0.001 mg/mL SM-EY extender. Different uppercase superscripts (A, B, and C) in the same row and lowercase superscripts (a and b) in the same column were significantly different (p < 0.05).

**Table 2:** The effect of nanoparticle green tea extract (NPs GTE) in skim milk-egg yolk (SM-EY) extender and different thawing temperatures on MDA level, SDF, and catalase levels of Kacang buck sperm.

**Parameters**	**Group**	**Thawing temperature**	
		**37°C**	**39°C**
MDA levels (nmol/mL)	Control	2275.28 ± 55.38^bB^	2059.42 ± 30.56^bA^
	NPs GTE	1619.24 ± 50.02^aB^	1416.96 ± 46.60^aA^
SDF (%)	Control	5.50 ± 0.55b	5.67 ± 0.52b
	NPs GTE	2.50 ± 0.55a	2.67 ± 0.82a
Catalase levels (x10^−3^ U/mg)	Control	56.27 ± 15.29	59.83 ± 15.01
	NPs GTE	54.12 ± 16.04	65.92 ± 10.75

Control: SM-EY extender, NPs GTE: addition of nanoparticles green tea extract 0.001 mg/mL SM-EY extender. Different uppercase superscripts (A, B, and C) in the same row and lowercase superscripts (a and b) in the same column were significantly different (p < 0.05).

Thawing temperature of 39ºC increases sperm viability, motility, and IPM and decreases MDA levels, compared with conventional thawing at 37ºC and decreased MDA levels than those thawed at 37°C (Table 2). However, SDF and catalase levels were similar (p>0.05) between thawing at 37ºC and thawing at 39ºC.

## Discussion

Nanomaterials can be applied to obtain bioactive properties of various elements in cell cryopreservation (Khalil et al., 2019). Antioxidant material in NPS forms a more effective play as ROS scavengers role, protecting membrane integrity from lipid peroxidation, thereby maintaining sperm viability and DNA integrity during cryopreservation (Falchi et al., 2018). In the NPS GTE, the content of Epigallocatechin Gallate (EGCG) in GTE has a high antioxidant effect that captures free radical scavenging, superoxide anion, and peroxy radicals, terminating the lipid peroxidation chain (Zhang et al., 2021), and phosphorylation of AMPK (Saeki et al., 2018). The supplementation of EGCG in the extender of frozen semen increases post-thawing sperm motility of buffalo (Ahmed et al., 2020), and bovine spermatozoa (Li et al., 2022). Sperm motility is the primary indicator of post-thawed semen quality (INSA, 2014), and it is correlated to MDA, IPM viability and SDF (Susilowati et al., 2022). Our previous study reported that the supplementation of GTE increases Kacang buck sperm motility using the Computer-assisted Sperm Analyzer (CASA) evaluation (Mustofa et al., 2021). However, sperm motility evaluation in this study was using microscope slide evaluation, did not use the CASA method due to the sperm motility parameters assessment using CASA did not show any difference with microscope slide evaluation (Ratnawati and Luthfi, 2020).

## Post-thaw sperm quality

Membrane integrity is essential for sperm viability and sperm motility (Dutta et al., 2019). Excessive production of ROS derived from the semen freezing-thawing process causes the changes of protein, lipid, and carbohydrate in sperm membrane (Pereira et al., 2017), followed by fragile and lose its semi permeability. Furthermore, the changes in sperm membrane structure and function during the freeze-thawing process caused interference of membrane activity followed by the death of spermatozoa. Higher ROS also impairs the proteins of the axoneme and mitochondria, which results in the loss of sperm motility, although the spermatozoa were alive (Peris-Frau et al., 2020). Cryopreservation leads to cold shock on the spermatozoa, damages the polyunsaturated fatty acids and cholesterol (Lone, 2018), therefore resulting in decreased stability of sperm membrane (Bergstein-Galan et al., 2018), and produces higher MDA levels (Fujii and Imai, 2014). ROS causes lipid peroxidation, mitochondrial and DNA bases disruption (Le et al., 2019), the interchain disulfide bond opening in protamines (Esteves et al., 2020), destabilization of the DNA structure, and SDF (Ribas-Maynou and Benet, 2019). The ejaculate defense system dealing with ROS includes antioxidant enzymes such as catalase. However, the small volume of sperm cytoplasm complicated the transport of antioxidants into another part of the sperm (Bibov et al., 2018). The inadequacy of antioxidants to face oxidative stress during cryopreservation had several effects on reducing sperm viability, sperm motility, and sperm plasma membrane integrity.

Epigallocatechin Gallate in GTE is an antioxidant that proved decreased lipid peroxidation, carbonylation of protein, and DNA damage. The mean particle size of NPSs GTE ins this study (208.00 ± 26.33 nm) was smaller than the GTE's mean microparticle size (40.43-225.64 µm) (Zokti et al., 2016). Nanoparticles extract have a larger surface-area (of cell membrane) to volume (of nanoparticles) ratio, followed by a greater surface area for interaction between nanoparticles and cell membranes (Abdelnour et al., 2021). Thereby, the sperm membrane allows NPs GTE to penetrate sperm cell better than GTE microparticles. Nanoparticles antioxidant effectively increase semen quality (Falchi et al., 2018). Green tea polyphenols as an antioxidant can increase semen quality due to the capability of catechin to ward off excessive ROS (Rahman et al., 2018). The presence of antioxidants in the extender promote a reduction in lipid peroxidation of sperm and, consequently, an increase in sperm viability, motility, and acrosome integrity (Martin-Hidalgo et al., 2019). Supplementation of 0.001% NPs GTE in semen extender increased post-thawed sperm quality. This result was according to the previous report that GTE's supplementation to a extender maintained the sperm motility, sperm viability, IPM, and sperm DNA integrity in post-thawed of Simmental bull semen (Susilowati et al., 2021b) and boar semen (Gale et al., 2015). The polyphenol groups of catechin in GTE have antioxidative activity and chelating agents. Meanwhile, its flavonoids act as stabilizers on lipid bilayers and membrane function (Roychoudhury et al., 2017). GTE polyphenol improves sperm quality through signaling pathways of Ferro-ferric iron to reduce ROS levels and prevent oxidation of low-density lipoproteins (Rahman et al., 2018), and signaling pathways of AMP-activated protein kinase, cAMP, calcium ion that prevent sperm DNA fragmentation (Selvam and Agarwal, 2018).

Semen has antioxidants themselves, i.e., glutathione peroxidase, superoxide dismutase, and catalase (Bibov et al., 2018). The superoxide dismutase activity is completed by catalase, which reduces hydrogen peroxide into water and molecular oxygen (Feng et al., 2020), thereby blocking the pathways that generate ROS and decreasing oxidative stress. The decrease in oxidative stress followed by increased membrane fluidity and percentage decrease in acrosome damages is due to membrane lipids and proteins rearrangements (Gungor et al., 2018). There are no reports on post-thawed levels of Kacang goat catalase yet. The supplementation of NPs GTE 0.001 mg/mL SM-EY extender caused no changes in the catalase levels compared to the control group. The result conforms to the report of Papas et al. (2019a) that the catalase activities were similar between good and poor freezability ejaculates. No correlation was found between the catalase activity in stallion seminal plasma and sperm parameters (Papas et al., 2019b). Catalase is produced by accessory glands and is present in seminal plasma, which functions as an antioxidant defense, including to the rise of ROS due to the freeze-thawing process (Pintus and Ros-Santaella, 2021). The same amount of ROS faced by same amount of catalase in both of the control group and the GTE NPs group. As previously mentioned, the nano size of GTE NPs makes it easier to enter sperm cells (Behzadi et al., 2017), thereby, it is suspected that NPs GTE not related to catalase levels measured in the extender. Further research is needed to prove this phonomenon.

## The thawing temperatures

Kacang goat frozen semen was better thawed at 39°C for 30 s. There is some variation in temperature and duration of thawing among different species. The ram’s sperm motility, viability, the functional and structural of the plasma membrane integrity were higher when straws were thawed at 39°C for 120 s or 50°C for 30 s compared with 90°C for 2 s, 75°C for 5 s, or 75°C for 10 s (Nicolae et al., 2014). Traditionally, goat frozen semen straw is thawed in a water bath at 37°C for 12-30 s (Narwade et al., 2017; Sharma et al., 2018; Sharma and Sood, 2019). The combination of duration and temperature of thawing for 45 s at 40°C (Ramachandran et al., 2015), for 20 s at 40°C (Sharma et al., 2018) have been studied with various results. Thawing of frozen semen will reactivate spermatozoa physiologically; hence, thawing has to be done carefully at an optimal temperature with sufficient time to minimize the decrease in semen quality (Borah et al., 2015). The thawing at 37 °C 20 s to 70 °C 12 s has not affected the midpiece and sperm head morphometry (Demirhan et al., 2020). However, the thawing of semen decreases the plasma membrane integrity, causes an early acrosomal reaction, and reduces sperm quality (Zenteno et al., 2023). Higher thawing temperature than optimum increased acrosomal damage, a rise in sperm metabolic rate (Borah et al., 2015), mitochondrial activity to produce more ATP, and the consequence is higher ROS production (Khalil et al., 2018), followed by hyperactivity and induce sperm death (Zenteno et al., 2023). In this study, the temperature of 39ºC result in an improvement in sperm quality than thawing at 37ºC. It may be due to 39ºC is the body temperature (Hereng et al., 2019; Sejian et al., 2021) and vagina temperature in estrus goat (Santoso et al., 2014).

## Conclusion

Supplementation of 0.001 mg/mL of NPs GTE in SM-EY extender resulted in higher post-thawed sperm viability, progressive motility, IPM, and lower MDA and SDF levels. Thawing temperature of 39°C resulted in a better quality of frozen-thawed Kacang goat semen.
